# T4-Locally Advanced Nasopharyngeal Carcinoma: Prognostic Influence of Cranial Nerve Involvement in Different Radiotherapy Techniques

**DOI:** 10.1155/2013/439073

**Published:** 2013-12-09

**Authors:** Hsin-I Huang, Kee-Tak Chan, Chih-Hung Shu, Ching-Yin Ho

**Affiliations:** ^1^Department of Otolaryngology, Taipei Veterans General Hospital, No. 201, Section 2, Shih-Pai Road, Taipei City 11217, Taiwan; ^2^School of Medicine, National Yang-Ming University, No. 201, Section 2, Shih-Pai Road, Taipei City 11217, Taiwan

## Abstract

*Background*. Cranial nerve involvement at disease presentation of nasopharyngeal carcinoma was not uncommon. We investigated the prognosis of patients with T4-locally advanced NPC, with or without cranial nerve involvement, and compared the outcome of patients treated using different radiotherapy techniques. *Methods*. In this retrospective study, 83 T4-locally advanced NPC patients were diagnosed according to the seventh edition of the American Joint Committee on Cancer staging system. All patients were treated using three-dimensional conformal radiotherapy (3D-CRT) or intensity-modulated radiation therapy (IMRT). The survival rate was analyzed using the Kaplan-Meier method. *Results*. The 5-year overall, locoregional-free, and disease-free survival rates of patients treated using IMRT were 88.9%, 75.2%, and 69.2%, respectively. The outcome in these patients was significantly better than that in patients treated using 3D-CRT, with survival rates of 58.2%, 54.4%, and 47.2%, respectively. There was no significant difference in the 5-year overall, locoregional-free, and disease-free survival rates of the patients with (64.2%, 60.5%, and 53.5%, resp.) and without (76.9%, 63.6%, and 57.6%, resp.) cranial nerve involvement. *Conclusion*. Locally advanced NPC patients treated using IMRT had significantly better outcomes than patients treated using 3D-CRT. Our results showed that the outcome of T4 NPC patients with or without cranial nerve involvement was not different.

## 1. Introduction 

Nasopharyngeal carcinoma (NPC), a tumor arising from the epithelial cells of the nasopharynx, is one of the most commonly diagnosed head and neck malignancies in Taiwan, with an annual incidence rate of 6.88 per 100,000 in 2007 [1, 2]. Because of the anatomic location of the nasopharynx and its tumor biology, radiotherapy-based treatment for nasopharyngeal carcinoma is the standard treatment modality [3, 4]. For early-stage nasopharyngeal carcinoma, the mainstay treatment is radiotherapy alone, and for advanced nasopharyngeal carcinoma, concomitant and neoadjuvant chemotherapy are suggested [3, 5]. Radiotherapy treatment remains challenging due to the proximity of the tumor to the surrounding vital organs, especially in tumors with intracranial extension [5–7]. Over the past decades, the development of three-dimensional conformal radiotherapy (3D-CRT) permits a more selective delivery than conventional radiotherapy. More recently, intensity-modulated radiation therapy (IMRT) produces more accurate dose distribution around targets [8–10].

Because of a rich submucosal lymphatic drainage system, early development of cervical lymph node metastasis occurs frequently and locoregional invasion and metastatic spread have prognostic value. The local failure rate correlates with advanced T stage [4]. Other important factors include the presence of cranial nerve palsy, skull base erosion, and oropharyngeal and parapharyngeal extensions [4, 11].

Approximately 70% of patients with NPC presents with locally advanced disease such as nonmetastatic stage III or IV disease [6]. According to the seventh edition of the American Joint Committee on Cancer (AJCC) staging system in 2010, nasopharyngeal tumors with intracranial extension and/or involvement of cranial nerves, hypopharynx, or orbit or those with extension to the infratemporal fossa or masticator space are defined as stage T4 [12]. Destruction of the skull base resulting in intracranial extension with cranial nerve involvement is not unusual because the cranial nerve is located adjacent to the skull base and the tumor is infiltrating in nature. It has been shown in previous studies that 11–29% patients had cranial nerve involvement at disease presentation [7, 13, 14]. The majority of cases with cranial nerve involvement are caused by superior invasion through the skull base into the cavernous sinus. The most commonly affected cranial nerve is the abducens nerve, followed by the trigeminal nerve. Many investigators have reported that cranial nerve deficit is a poor prognostic factor in T4 tumors [4, 7, 15]. However, most of these studies used the American Joint Committee on Cancer (AJCC) staging system prior to 1997, which also classified cases with skull base erosion as stage T4.

The purpose of this study was to analyze the outcome of nonmetastatic T4 NPC patients treated at our department between January 1997 and January 2007. We compared the outcomes of patients treated using 3D-CRT and IMRT and the effect of cranial nerve involvement.

## 2. Methods

Between January 1997 and January 2007, 879 new NPC patients were diagnosed in the Department of Otolaryngology, Taipei Veterans General Hospital, Taiwan. Patients who had distant metastasis or disrupted treatment were excluded from this study. Eighty-three patients (9.4%) were diagnosed with T4-locally advanced disease and were enrolled in our study. The study was approved by the hospital's Institutional Review Board (IRB 2012-02-020AC). All patients underwent pretreatment evaluation, including complete medical history, physical and neurological examination; hematology and biochemistry profiles; and chest radiography, abdominal sonography, whole body bone scan, and magnetic resonance imaging (MRI) of the head and neck. They were restaged according to the seventh edition of the AJCC classification system. All patients were treated using external radiotherapy with or without concomitant or neoadjuvant cisplatin-based chemotherapy.

Follow-up data were collected at periodic visits to our clinic until January 2012. The follow-up period was considered as the duration from the day of the first treatment to the day of death or the last clinic visit before analysis.

Statistical analyses were performed using the Statistical Package for the Social Sciences (SPSS) 19.0 software. The between-groups analysis was calculated using Chi-Square test. The survival rate was calculated using the Kaplan-Meier method. *P* value of less than 0.05 was considered statistically significant.

## 3. Results

### 3.1. Patient Distribution

Data from 83 patients were collected and were analyzed retrospectively in our study. There were 69 (83.1%) men and 14 (16.9%) women, with a mean age of 50.8 ± 14.0 years (range, 18–78 years). The mean follow-up period was 66.5 months (range, 1–174 months). Fifty-three patients (63.9%) received 3D-CRT and 30 (36.1%) received IMRT. Neoadjuvant or concomitant cisplatin-based chemotherapy was administered to 32 patients (60.4%) in 3D-CRT group and 25 patients (83.3%) in IMRT group. The age, sex, and N status between 3D-CRT group and IMRT group showed no significant difference. Patients with cranial nerve involvement were 42 patients (77.4%) and 13 patients (43.3%) in 3D-CRT group and IMRT group, respectively. It was significantly high in 3D-CRT group, as shown in Table 1.

The most common symptoms of T4-staged nasopharyngeal carcinoma were diplopia (22.9%), followed by headache (15.7%), aural symptoms (14.5%), and neck mass (12.0%). Cranial nerve involvement was seen in 65.1% (54/83) of the cases. Seventeen of these patients showed involvement of multiple cranial nerves. The most commonly involved cranial nerve was the cranial nerve VI (63.0%), followed by the cranial nerve V (44.4%), II (13.0%), III (9.3%), and X (5.6%). Based on the level of cranial nerve involvement, the patients were divided into 2 groups: the anterior group, which includes cranial nerves from I to VIII, and the posterior group, which includes cranial nerves from IX to XII. Of the 54 patients with cranial nerve paralysis, 50 showed involvement of the anterior cranial nerves, 3 showed involvement of the posterior cranial nerves, and only 1 showed involvement of both the anterior and the posterior cranial nerves.

### 3.2. Survival Analysis

Of the 53 cases treated using 3D-CRT, 44 (83.0%) were men and 9 (17.0%) were women. Twelve patients (22.6%) were staged as N0, 17 (32.1%) as N1, 23 (43.4%) as N2, and 1 (1.9%) as N3. Of the 30 cases treated using IMRT, 25 (83.3%) were men and 5 (16.7%) were women. Three patients (10.0%) were staged as N0, 7 (23.3%) as N1, 19 (63.3%) as N2, and 1 (3.3%) as N3. Patient's sex and nodal stage did not significantly affect the survival rate in the 3D-CRT or IMRT groups.

**Figure 1:**
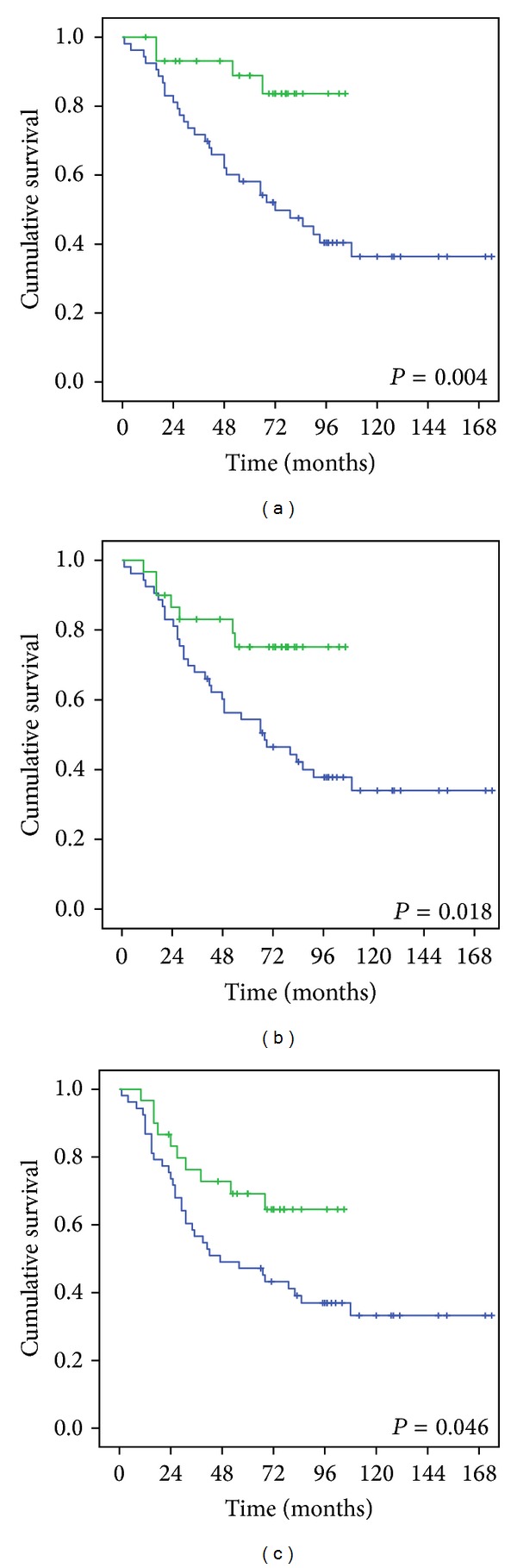
Comparison of (a) the 5-year overall survival, (b) the 5-year locoregional-free survival, and (c) the 5-year disease-free survival in T4-locally advanced NPC patients treated using IMRT (green line) or using 3D-CRT (blue line).

**Figure 2:**
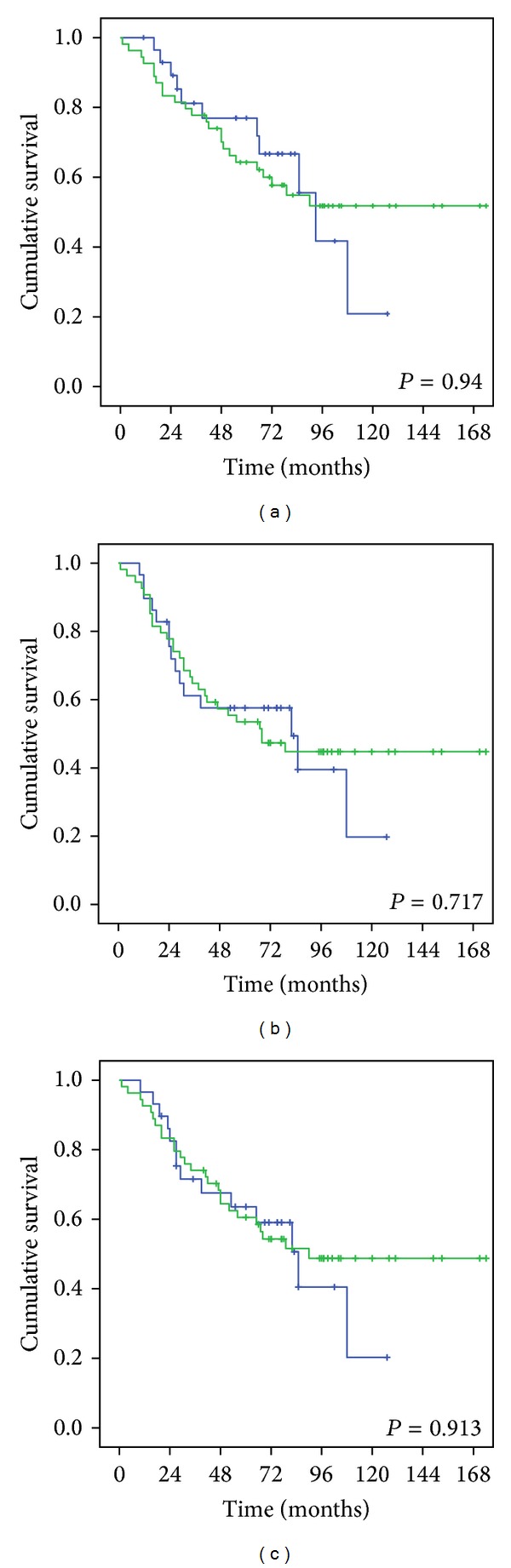
Comparison of (a) the 5-year overall survival, (b) the 5-year locoregional-free survival, and (c) the 5-year disease-free survival in T4-locally advanced NPC patients with cranial nerve involvement (green line) or without cranial nerve involvement (blue line).

The 5-year overall, locoregional-free, and disease-free survival rates of patients treated using IMRT were 88.9%, 75.2%, and 69.2%, respectively. These results were significantly better than those in patients treated using 3D-CRT, who had survival rates of 58.2%, 54.4%, and 47.2%, respectively (*P* = 0.004, 0.018, and 0.046, resp.) (Figure 1). The 5-year overall, locoregional-free, and disease-free survival rates were 64.2%, 60.5%, and 53.5%, respectively, in patients with cranial nerve involvement and were 76.9%, 63.6%, and 57.6%, respectively, in patients without cranial nerve involvement. There was no statistical difference for these values between the 2 groups (*P* = 0.94, 0.717, and 0.913, resp.) (Figure 2). Furthermore, the survival rates of patients with involvement of anterior, posterior, or both anterior and posterior cranial nerves showed no significant difference.

**Figure 3:**
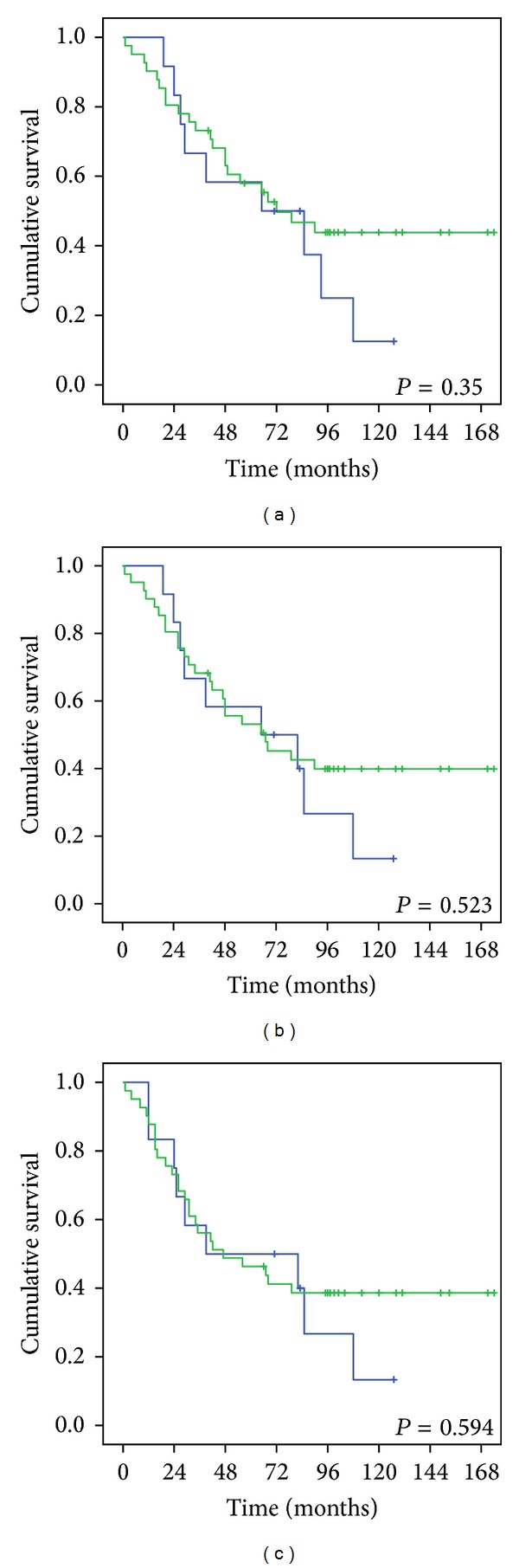
Comparison of (a) the 5-year overall survival, (b) the 5-year locoregional-free survival, and (c) the 5-year disease-free survival in the 3D-CRT group with cranial nerve involvement (green line) or without cranial nerve involvement (blue line).

**Figure 4:**
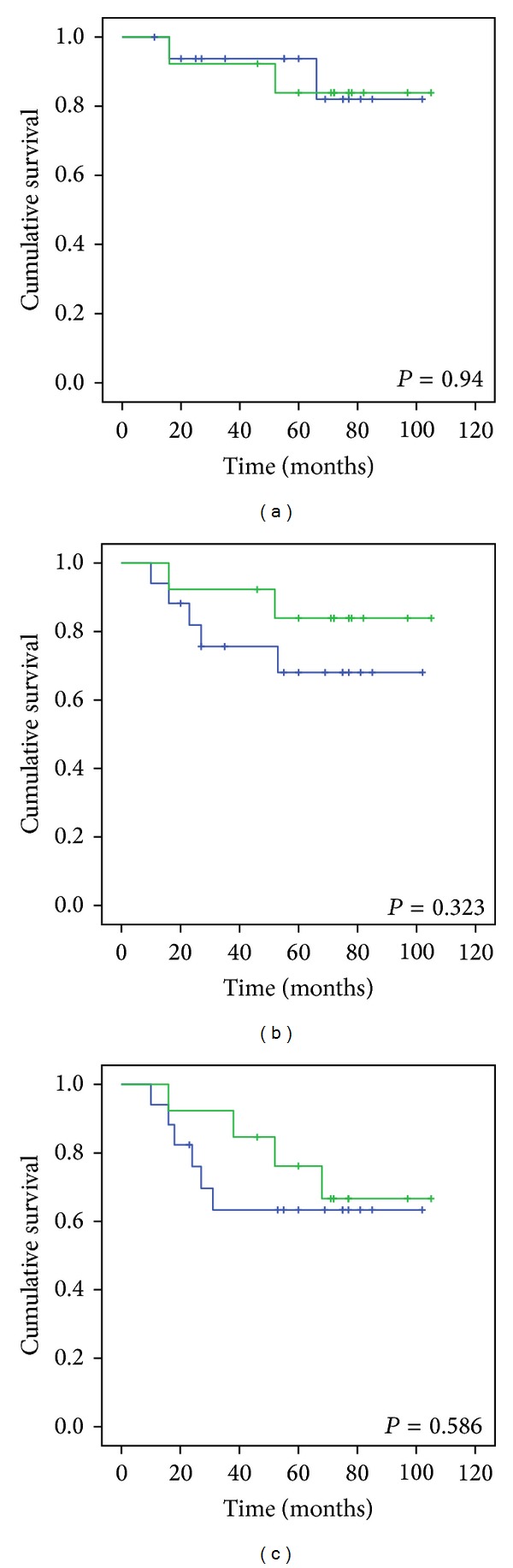
Comparison of (a) the 5-year overall survival, (b) the 5-year locoregional-free survival, and (c) the 5-year disease-free survival in the IMRT group with cranial nerve involvement (green line) or without cranial nerve involvement (blue line).

We also divided these 83 patients into 2 groups: one group underwent 3D-CRT therapy and the other underwent IMRT therapy. The 5-year overall, locoregional-free, and disease-free survival rates of patients in the 3D-CRT group with cranial nerve involvement were 58%, 53.1%, and 46.3%, respectively, and in those without cranial nerve involvement were 58.3%, 58.3%, and 50%, respectively (*P* = 0.35, 0.523, and 0.594, resp.) (Figure 3). The corresponding values for patients in the IMRT group with cranial nerve involvement were 83.9%, 83.9%, and 76.2%, respectively, and in those without cranial nerve involvement were 93.8%, 68.1%, and 63%, respectively (*P* = 0.94, 0.323, and 0.586, resp.) (Figure 4). Therefore, cranial nerve involvement did not influence the overall five-year survival of patients in the 3D-CRT or IMRT groups.

## 4. Discussion

In our experience, compared to 3D-CRT, IMRT in locally advanced NPC patients showed significantly better results. We also found that cranial nerve involvement did not influence the overall five-year survival in patients with T4-locally advanced NPC.

Radiation-based therapy has been considered the standard modality for treating NPC patients [3, 4]. Locoregional control is a fundamental goal of NPC treatment, and locoregional recurrence has been associated with poor outcome and a high risk of distant metastasis [4, 11]. However, approximately 70% of patients present with locally advanced nonmetastatic disease [6]. Various radiotherapy techniques have been introduced in an attempt to improve the locoregional control of NPC using primary radiotherapy while reducing toxicity to normal organs. The development of IMRT has gained popularity for the treatment of head and neck cancer, including nasopharyngeal carcinoma [8–10]. With this technique, the intensity of the radiation beams can be modulated such that a high dose can be delivered more accurately to the target tumor while significantly reducing the dose to the surrounding vital organs and normal tissues [16]. The IMRT technique has gradually replaced conventional radiotherapy for the treatment of NPC as a standard treatment modality, because it delivers higher radiation dose to the primary disease and neck metastases while sparing the organs at risk, thereby enhancing the therapeutic ratio [8, 9, 16, 17].

Radiotherapy for patients with NPC is challenging because it requires delivery of an adequate dose to the target tumor without causing potentially serious complications to adjacent critical organs, especially in patients with cranial nerve involvement and intracranial extension [16, 17]. We found that the 5-year overall, locoregional-free, and disease-free survival rates of T4-locally advanced NPC patients treated using IMRT were 88.9%, 75.2%, and 69.2%, respectively, which were significantly better than the corresponding values (58.2%, 54.4%, and 47.2%, resp.) in patients treated using 3D-CRT (*P* = 0.004, 0.018, and 0.046, resp.).

Most of studies documented N status as a prognostic factor for survival. Liu et al. reported that T stage of disease was a significant predictor of disease-free survival, favoring those with early-stage (T1-2) disease, and that N status was also a significant prognostic factor for the overall survival [4]. Lee et al. found that patients with more aggressive N statuses have poorer clinical outcomes, but the influence was smaller in T4-staged patients [18]. However, we found that N status does not affect the survival rates. All of our patients were diagnosed with T4 disease. Although our N3 group was small, we found that N status had less influence in patients with advanced primary tumor.

It has been shown, that compared to conventional radiotherapy, IMRT better improves the outcome of nasopharyngeal carcinoma [8, 9, 16]. Özyar et al. reported 3-year overall survival rates of 71 and 60% and disease-free survival rates of 74 and 46% for IVA- and IVB-staged patients, respectively. Their results also showed that advanced N status was an unfavorable prognostic factor both for overall (*P* = 0.03), disease-free (*P* = 0.0004), and distant metastasis-free (*P* = 0.0003) survival [11]. Lai et al. observed a trend of improvement in disease-free survival in the IMRT group compared to the two-dimensional radiotherapy (2DRT) group [16]. A study at the Memorial Sloan-Kettering Cancer Center also found a trend for improved local control with IMRT compared to local control of 79% in 35 patients treated using 3D-CRT (*P* = 0.11) [10]. Excellent locoregional control for NPC was also achieved using IMRT in a University of California, San Francisco, study. The estimated 4-year local progression-free, locoregional progression-free, and distant metastases-free rates were 97%, 98%, and 66%, respectively, and the 4-year overall survival was estimated to be 88% [9]. Our data confirmed that the local and distant disease control in locally advanced NPC was better by using IMRT than 3D-CRT.

The diagnosis of nasopharyngeal carcinoma can be a challenge to physicians. This is because nasopharyngeal neoplasm may hide all nasal and aural symptoms and present nonspecific signs such as diplopia, facial numbness, or headache as the initial manifestation [2, 19]. Eleven to 29% patients showed cranial nerve involvement at disease presentation [7, 13, 14]. Cranial nerve involvement was observed in 65.1% of our nonmetastatic T4 NPC patients. The abducens and the trigeminal nerves were the most frequently affected. Based on cranial nerve involvement, our patients were classified into 2 subgroups. The 5-year overall, locoregional-free, and disease-free survival rates of patients with cranial nerve involvement were 64.2%, 60.5%, and 53.5%, respectively, and in those without cranial nerve involvement were 76.9%, 63.6%, and 57.6%, respectively. There were no significant differences in these values between the 2 groups. We also divided the level of cranial nerves into anterior and posterior groups and the survival rates did not differ between these groups. Furthermore, we found that there was no significant difference in the 5-year overall, locoregional-free, and disease-free survival rates between patients with T4 disease with or without cranial nerve involvement in the 3D-CRT group (58%, 53.1%, and 46.3%, resp., versus 58.3%, 58.3%, and 50%, resp.; *P* = 0.35, 0.523, and 0.594, resp.) or the IMRT group (83.9%, 83.9%, and 76.2%, resp., versus 93.8%, 68.1%, and 63%, resp.; *P* = 0.94, 0.323, and 0.586, resp.).

Roh et al. investigated prognostic factors in nasopharyngeal carcinoma and found that patients with involvement of both anterior and posterior cranial nerves had a worse prognosis than those with involvement of either anterior or posterior cranial nerves (*P* = 0.0219) [15]. Chang et al. also presented similar results. They found that patients with extensive cranial nerves involvement have worse survival than patients with limited involvement of anterior or posterior cranial nerves (*P* < 0.001) [20]. Our data showed similar survival in the anterior group, the posterior group, and the group with involvement of both anterior and posterior nerves. But a majority of patients in this study had only anterior cranial nerve involvement (92.6%). Further studies are needed to establish the role of different groups of cranial nerve involvement in NPC survical.

Cooper et al. found that the outcome in subgroups of T4-locally advanced NPC disease was not significantly different based on cranial nerve involvement alone, skull base erosion alone, or both. In most studies, cranial nerve involvement was recognized as a poor prognostic factor [21]. Altun et al. reported that the overall 5-year survival rate in patients with cranial nerve deficit was 25% compared to 58% in patients without cranial nerve deficit (*P* = 0.01). They documented that patients with cranial nerve palsy had a worse prognosis than patients with skull base erosion alone [13]. We restaged all patients according to the seventh edition of the AJCC staging manual in 2010. Our data showed that cranial nerve involvement did not affect the prognosis of T4-locally advanced NPC patients. It is important to note that previous reports used an older staging system, which classified skull base invasion as a T4 stage. According to the current staging system, skull base destruction is defined as T3 stage, which has a better prognosis than T4 stage.

Limitations of this study include a small sample size in the IMRT group because our medical center started using IMRT for the treatment of NPC patients only in late 2003. A larger population of patients and a longer follow-up period to evaluate the long-term outcomes and complications are needed. Due to its retrospective nature, chemotherapy in our studies was not uniform. In addition, cranial nerve involvement may be asymptomatic, and sometimes the symptoms may be subtle. Evaluation of cranial nerve palsy by using clinical symptoms and physical examination also has certain limitation; therefore, a more accurate and careful neurological examination is required.

## 5. Conclusion

In conclusion, we found that cranial nerve involvement, which was proposed to be a poor prognostic factor in the past, had no significant effect on the survival of T4-locally advanced NPC patients. Patients with locally advanced NPC should be encouraged to complete the entire course of treatment. IMRT delivers higher radiation dose and a better coverage of the tumor region thereby enhancing the therapeutic ratio. Improvement of treatment modality, better radiotherapy technique combined with chemotherapy, increased the survival rate of locally advanced NPC patients.

## Figures and Tables

**Table 1:** Patients characteristics in different radiotherapy techniques (*n* = 83).

	3D-CRT (*n* = 53)		IMRT (*n* = 30)		
	Number	Percentage (%)	Number	Percentage (%)	
Age	Mean: 52.3 ± 13.9		Mean: 48.2 ± 14.0		*P* = 0.14
	(range: 18–78)		(range: 19–78)	
Sex					
Male	44	83.0	25	83.3	*P* = 0.97
Female	9	17.0	5	16.7	
N stage					
N0	12	22.6	3	10.0	*P* = 0.28
N1	17	32.1	7	23.3	
N2	23	43.4	19	63.3	
N3	1	1.9	1	3.3	
Chemotherapy					
Yes	32	60.4	25	83.3	*P* = 0.03
No	21	39.6	5	16.7	
CN involvement					
Yes	41	77.4	13	43.3	*P* = 0.002
No	12	22.6	17	56.7	

*P* value of <0.05 indicates statistical significance.

3D-CRT: three-dimensional conformal radiotherapy; IMRT: intensity-modulated radiotherapy; CN: cranial nerve.
